# Health care cost associated with the use of enzyme-inducing and non-enzyme–active antiepileptic drugs in the UK: a long-term retrospective matched cohort study

**DOI:** 10.1186/s12883-017-0837-y

**Published:** 2017-03-23

**Authors:** Simon Borghs, Solène Thieffry, Matthias Noack-Rink, Peter Dedeken, Lai San Hong, Laura Byram, John Logan, Jane Chan, Victor Kiri

**Affiliations:** 1 UCB Pharma, Slough, UK; 2 UCB Pharma, Brussels, Belgium; 3 UCB Pharma, Monheim Am Rhein, Germany; 4 Redsen Limited, Bournemouth, UK; 5 Stats4Pharma, Cork, Ireland; 6 FV & JK Consulting Ltd., Guildford, UK

**Keywords:** Epilepsy, Health care costs, Cytochrome P450 enzyme system, Drug-related side effects and adverse reactions, Comorbidity, Database, Hospital records

## Abstract

**Background:**

Some antiepileptic drugs (AEDs) induce expression of hepatic enzymes. This can contribute to comorbidities via interference with metabolic pathways and concomitant drug metabolization, thereby increasing the likelihood of health care interventions. Using medical records, we compared the direct health care cost in patients initiating epilepsy therapy with enzyme-inducing AEDs (EIAEDs) vs non-enzyme-active AEDs (nEAAEDs) over up to 12 years.

**Methods:**

Patients with untreated epilepsy were indexed in the UK Clinical Practice Research Datalink and Hospital Episode Statistics database when prescribed a new EIAED or nEAAED between January 2001 and December 2010. Propensity score matching reduced confounding factors between cohorts. Patients were followed until cohort treatment failure or data cut-off. The primary outcome was the median standardized monthly direct health care cost during follow-up in 2014 £GBP, calculated using published reference costs and compared using a Mann–Whitney *U* test.

**Results:**

The unmatched EIAED cohort (*n* = 2752) was older (54 vs 46 years), more likely to be male, had more comorbidities, and higher health care resource use/cost during the 1-year pre-index period (median £3014 vs £2516) than the nEAAED cohort (*n* = 2,137). The most common index EIAED and nEAAED were carbamazepine (63.3%) and lamotrigine (58.0%), respectively. After matching, cohorts had similar features (*n* = 951 each). Over up to 12 years of follow-up, the median standardized monthly direct health care cost was £229 for the EIAED and £188 for the nEAAED cohorts (*p* = 0.0091). The median cost was higher for the EIAED cohort in every year of follow-up. In the two cohorts, 25.1% and 20.1% of total mean cost during follow-up was epilepsy-related, with approximately 4.6% and 3.0% for AED acquisition, respectively. The median time to cohort treatment failure was shorter in the matched EIAED cohort (468 vs 1194 days).

**Conclusions:**

Patients in the UK who initiated epilepsy therapy with an EIAED appeared to be at higher risk of complications associated with enzyme induction. In long-term matched cohort analyses, the median total direct health care cost associated with EIAED therapy was higher than with nEAAEDs. Changing current treatment practices could potentially improve patient outcomes and reduce costs.

**Electronic supplementary material:**

The online version of this article (doi:10.1186/s12883-017-0837-y) contains supplementary material, which is available to authorized users.

## Background

In 2010, the Joint Epilepsy Council of the UK and Ireland estimated that 0.97% of the UK population (~602,000 people) had epilepsy [1]. For many patients, epilepsy is a chronic disorder that requires long-term antiepileptic drug (AED) therapy. A broad array of AEDs is available, with varying mechanisms of action and pharmacokinetic profiles. Enzyme-inducing AEDs (EIAEDs), such as carbamazepine and phenytoin, stimulate the synthesis of endogenous cytochrome P450 (CYP450) enzymes as an off-target effect. Only a few CYP450 enzymes are responsible for approximately 80% of all oxidative drug metabolism [2]. Consequently, treatment with enzyme-inducing drugs can potentially lead to altered metabolization of some concomitant drugs, and requires a distinct set of treatment considerations [2–4]. Other AEDs, such as oxcarbazepine and topiramate (mild EIAEDs), induce only a few CYP450 enzymes, while valproate is a CYP450 inhibitor [2, 5].

Although non-enzyme-active AEDs (nEAAEDs) such as lamotrigine, zonisamide and levetiracetam are available [5], EIAEDs remain widely used in the treatment of epilepsy. No clear preference for EIAED or nEAAED prescribing is made in UK treatment guidelines, which are developed with consideration for the balance between clinical outcomes and cost [6].

It has previously been hypothesized that EIAED therapy could lead to higher long-term health care resource use and cost [2]. Firstly, patients prescribed EIAEDs may require higher dosages of concomitant drugs metabolized by the CYP450 system, with more frequent primary care appointments to monitor/titrate drug levels and manage potential side effects. Secondly, metabolic changes related to enzyme induction may contribute to the development of comorbidities such as osteoporosis, sexual dysfunction and vascular disease [2, 7, 8]. Additional investigation and treatment of these comorbidities may contribute to increased health care costs. Thirdly, discontinuation of EIAEDs may lead to increases in concomitant medication levels, requiring additional primary care appointments to monitor and manage any potential toxicity.

A recent literature review has shown that while costs associated with the use of specific AEDs have been reported, the costs associated with EIAED therapy vs other AED therapy have not previously been compared over a clinically meaningful time period [9]. Therefore, the objectives of this study were to compare the characteristics of patients prescribed EIAEDs and nEAAEDs, and to estimate and compare the all-cause direct health care cost associated with the use of EIAEDs vs nEAAEDs as epilepsy therapy in the UK over up to 12 years of follow-up.

## Methods

### Source data

This retrospective matched cohort study used primary care data included in the October 2014 update of Clinical Practice Research Datalink (CPRD-GOLD; The National Health Service National Institute for Health Research & The Medicines & Healthcare Products Regulatory Agency, London, UK), linked to admitted patient care data contained in the national Hospital Episode Statistics database (HES; Health and Social Care Information Centre, Leeds, UK). The study protocol was reviewed and approved by the Independent Scientific Advisory Committee (the scientific ethics committee concerned with the use of CPRD data).

### Patient selection and follow-up

The study consisted of two cohorts: patients prescribed an index EIAED (carbamazepine, phenytoin, phenobarbital or primidone) and those prescribed an index nEAAED (gabapentin, lacosamide, lamotrigine, levetiracetam, perampanel, pregabalin, retigabine, vigabatrin or zonisamide). All other AEDs were not considered index AEDs. Mild EIAEDs (eslicarbazepine acetate, oxcarbazepine, rufinamide or topiramate) were not allowed during the pre-index or follow-up periods. Given these AEDs’ mild enzyme induction profile, classifying them as either EIAEDs or nEAAEDs, or allowing their use in follow-up, would have potentially biased the comparison of cost outcomes between EIAEDs and nEAAEDs. Clonazepam and clobazam were not considered potential long-term therapies, and their use was permitted during the pre-index and follow-up periods.

Patients ≥ 16 years of age and diagnosed with epilepsy were selected from the CPRD database if they were first prescribed an EIAED or nEAAED (index AED) between January 1, 2001 and December 31, 2010 (the selection period). The index date was the time of first prescription of an index AED during the selection period. Patients could have been diagnosed on or at any time prior to the index date. For each selected patient, CPRD and HES data had to be available for the 1-year pre-index period. Patients had not previously used any index AED (i.e. any of the EIAEDs/nEAAEDs of interest) at any time during the available data coverage and had not received *any* AED treatment during the 1-year pre-index (i.e. baseline) period. Treatment with AEDs other than an index AED was permitted before the pre-index period, to avoid excluding patients who had used AEDs for non-epilepsy indications. At least 31 days of index AED exposure were required for inclusion, in order to include patients with short treatment durations, but at the same time exclude patients who might not have taken the index AED. Patients who were prescribed an index AED but remained registered in the CPRD for less than 31 days were nevertheless included, to avoid excluding patients who did take the index AED but for whom further prescription data to determine this is unavailable. Patients starting more than one AED on the index date were excluded.

Patients were followed until any of the following events occurred: end of primary care or HES data coverage, end of registration/death, cohort treatment failure (defined as addition of an AED belonging to the other cohort or discontinuation of all AED(s) belonging to the index cohort), or addition of a mild EIAED. Patients switching between/adding within-cohort AEDs (e.g. carbamazepine for phenytoin) remained in follow-up.

### Exposure

Exposure was defined using primary care prescription data. We used an algorithm to impute the maximum duration of individual AED prescriptions. The most likely maximum duration of each AED prescription was calculated as twice the median duration for all prescriptions of that type (type defined based on generic name, dose and package size) in the entire study database. Each day of patient follow-up was then categorised as exposed or not exposed using the prescription dates and imputed maximum durations. The end of exposure to an AED was defined as the start date of an exposure gap longer than four times the imputed maximum duration of the last prescription.

### Baseline characteristics

Patient demographics and clinical characteristics were identified using primary care data and read codes, and in HES, using the *International Classification of Disease (Tenth Revision)* codes. These were used to calculate a Germaine-Smith epilepsy comorbidity index score for each cohort [10]. Concomitant medications were identified through primary care prescription data.

### Health care resource use and costs by type

The costs associated with each health care resource used were estimated, per type, in 2014 £GBP, including any prescriptions (AEDs or otherwise), primary care consultations (general practitioner [GP] consultations and patient-related activity at the GP practice), test procedures, accident and emergency (A&E) visits, hospitalizations and outpatient referrals and procedures. Our costing protocol was partly based on principles previously applied in the literature [9]. Further details on the costing procedure can be found in the Additional file 1.

### Matching

Propensity score matching was used to reduce the effect of confounding factors between cohorts, a methodology commonly used in studies based on observational data [11]. EIAED and nEAAED patients were matched 1:1 on propensity score, where propensity score was defined as the probability of a patient being prescribed an EIAED. The propensity scores were derived using a multivariate logistic model. The choice of potential confounding variables used in the model was guided by the literature; variables were selected that were likely to influence treatment choice and affect the outcome. The estimation process used all available patient-level characteristics at baseline that had a minimum incidence of 1.0%, or were significantly different between the two unmatched cohorts when compared by either a two-sample *t* test, chi-square test or Fisher’s exact test, as suitable. Table S1 shows variables that were found to be significantly different between the cohorts prior to propensity score matching. Patients were only included in the matched analysis if a suitable pairing was found.

### Study outcomes and statistical analyses

To compare the characteristics of patients prescribed EIAEDs and nEAAEDs, baseline characteristics in the unmatched cohorts were compared using unpaired, two-sample *t* tests for continuous variables and chi-square tests for categorical variables. To assess propensity score matching success, baseline characteristics in the matched cohorts were compared using the same procedures.

To calculate the primary endpoint, the total health care cost per patient was summed over the follow-up period and divided by the number of months of patient follow-up, thus arriving at the standardized monthly direct health care cost during follow-up. Medians were compared between the matched cohorts using a Mann–Whitney *U* test. The median is preferred to the mean because of the highly skewed distribution of the health care cost variable. Standardized monthly cost per year was calculated in a similar manner, by summing costs for the year and dividing by the number of months of patient follow-up for that year. In addition, the cost and incidence of specific health care resources used during follow-up for the matched cohorts were split by type; the costs and incidence of epilepsy-related resource use were estimated; the incidence of new non-AED medication use and (non-epilepsy) comorbid diagnoses during follow-up were described for the matched cohorts; and the time to cohort treatment failure and to index AED treatment failure were compared between the matched cohorts using Kaplan-Meier methods, censoring patients reaching the end of follow-up. Index AED failure was defined as the end of exposure to the AED prescribed at index or addition of any other AED, whichever occurred earlier. Index AED failure differs from cohort treatment failure in that it does not allow within-cohort AED switching or add-on.

Analyses were performed using SAS 9.3 (SAS Institute, Cary, NC, USA). No formal power calculations for sample size were performed.

## Results

### Baseline characteristics in the 1-year pre-index period

In the overall population, 4889 unmatched patients were indexed. Patients in the EIAED cohort (*n* = 2752) were older, more likely to be male and had a higher mean baseline Germaine-Smith epilepsy comorbidity index score than those in the nEAAED cohort (*n* = 2137; *p* < 0.0001; Table 1). More patients with index EIAEDs had pre-index diagnoses of cardiovascular disease (EIAED 26.1% vs nEAAED 17.5%), hypertension (15.1% vs 11.3%) and neoplasms (11.4% vs 6.0%). A lower proportion of patients in the EIAED cohort received hormonal contraceptives (3.5% vs 11.9% in the nEAAED cohort) and antidepressant/antipsychotic drugs (27.4% vs 33.7%) in the 1-year pre-index period. The largest proportion of patients entered the EIAED cohort in 2004 (14.6%; Table 2), decreasing in subsequent years. Conversely, nEAAED cohort entry steadily increased over the selection period, peaking in 2010 (17.8%; Table 2). Carbamazepine was the most commonly prescribed index EIAED (63.3%), and lamotrigine the most common index nEAAED (58.0%) (Table 2).

**Table 1:** Baseline demographics and epilepsy characteristics in the 1-year pre-index period

Characteristic	Overall population			Matched population		
	EIAED (*n* = 2752)	nEAAED (*n* = 2137)	*p* value^a^	EIAED (*n* = 951)	nEAAED (*n* = 951)	*p* value^a^
Age, mean (SD), years	54.2 (19.8)	45.9 (19.9)	<0.0001	47.7 (19.9)	48.0 (20.0)	0.7969
Age group, *n* (%)			<0.0001			1.0000
16–20	116 (4.2)	239 (11.2)		75 (7.9)	75 (7.9)	
21–30	261 (9.5)	349 (16.3)		144 (15.1)	144 (15.1)	
31–40	404 (14.7)	344 (16.1)		167 (17.6)	167 (17.6)	
41–50	434 (15.8)	380 (17.8)		160 (16.8)	160 (16.8)	
51–60	406 (14.8)	285 (13.3)		133 (14.0)	133 (14.0)	
61–70	425 (15.4)	236 (11.0)		119 (12.5)	119 (12.5)	
71–80	434 (15.8)	191 (8.9)		94 (9.9)	94 (9.9)	
81–90	237 (8.6)	96 (4.5)		47 (4.9)	47 (4.9)	
Over 90	35 (1.3)	17 (0.8)		12 (1.3)	12 (1.3)	
Gender, *n* (%)			<0.0001			0.7130
Female	1243 (45.2)	1339 (62.7)		506 (53.2)	514 (54.0)	
Male	1509 (54.8)	798 (37.3)		445 (46.8)	437 (46.0)	
Germaine-Smith epilepsy-specific comorbidity index, mean score (SD)	1.0 (2.0)	0.6 (1.6)	<0.0001	0.7 (1.6)	0.7 (1.5)	0.8272
Time since first epilepsy diagnosis, years						
Mean (SD)	4.5 (10.3)	8.5 (13.5)	<0.0001	5.8 (11.1)	6.2 (12.0)	0.4604
Median (P25–P75)	0.2 (0.1–2.5)	1.3 (0.1–10.9)		0.4 (0.1–5.8)	0.6 (0.1–6.3)	
Epilepsy type, *n* (%)			0.0011			0.7202
Generalized	301 (10.9)	212 (9.9)		78 (8.2)	85 (8.9)	
Partial	470 (17.1)	291 (13.6)		148 (15.6)	138 (14.5)	
Unspecified	1981 (72.0)	1634 (76.5)		725 (76.2)	728 (76.6)	

*Abbreviations: EIAED* enzyme-inducing antiepileptic drug, *nEAAED* non-enzyme–active antiepileptic drug, *P25–P75* 25th to 75th percentile, *SD* standard deviation

^a^ *t* test for continuous variables, chi-square test for categorical variables

**Table 2:** Index year and AED

	Overall population			Matched population		
	EIAED (*n* = 2752)	nEAAED (*n* = 2137)	*p* value^a^	EIAED (*n* = 951)	nEAAED (*n* = 951)	*p* value^a^
Year of index, *n* (%)			<0.0001			0.0669
2001	298 (10.8)	70 (3.3)		45 (4.7)	55 (5.8)	
2002	326 (11.8)	88 (4.1)		53 (5.6)	63 (6.6)	
2003	312 (11.3)	121 (5.7)		86 (9.0)	72 (7.6)	
2004	402 (14.6)	147 (6.9)		109 (11.5)	85 (8.9)	
2005	314 (11.4)	190 (8.9)		106 (11.1)	96 (10.1)	
2006	275 (10.0)	222 (10.4)		121 (12.7)	109 (11.5)	
2007	256 (9.3)	255 (11.9)		121 (12.7)	119 (12.5)	
2008	225 (8.2)	293 (13.7)		106 (11.1)	123 (12.9)	
2009	206 (7.5)	371 (17.4)		130 (13.7)	119 (12.5)	
2010	138 (5.0)	380 (17.8)		74 (7.8)	110 (11.6)	
Index AED, *n* (%)			<0.0001			<0.0001
EIAED cohort						
Carbamazepine	1742 (63.3)	—		690 (72.6)	—	
Phenytoin	971 (35.3)	—		245 (25.8)	—	
Phenobarbital	26 (0.9)	—		9 (0.9)	—	
Primidone	13 (0.5)	—		7 (0.7)	—	
nEAAED cohort						
Lamotrigine	—	1239 (58.0)		—	632 (66.5)	
Gabapentin	—	448 (21.0)		—	149 (15.7)	
Levetiracetam	—	261 (12.2)		—	126 (13.2)	
Pregabalin	—	185 (8.7)		—	41 (4.3)	
Zonisamide	—	2 (0.1)		—	2 (0.2)	
Vigabatrin	—	2 (0.1)		—	1 (0.1)	

*Abbreviations: AED* antiepileptic drug, *EIAED* enzyme-inducing antiepileptic drug, *nEAAED* non-enzyme–active antiepileptic drug

^a^Chi-square test

During the 1-year pre-index period, patients in the unmatched EIAED cohort had a higher all-cause direct health care cost and a higher mean epilepsy-related health care cost than those in the unmatched nEAAED cohort (Table 3). Patients in the EIAED cohort had higher utilization of acute health care resources during the 1-year pre-index period; a reflection of higher mean numbers of A&E visits and inpatient hospitalizations per patient (Table 3). However, the mean numbers of GP consultations and outpatient hospitalizations were higher in the nEAAED cohort (Table 3).

**Table 3:** Health care costs and resource use in the 1-year pre-index period

	Overall population			Matched population		
	EIAED (*n* = 2752)	nEAAED (*n* = 2137)	*p* value^a^	EIAED (*n* = 951)	nEAAED (*n* = 951)	*p* value^a^
All-cause direct health care costs in the 1-year pre-index period, £						
Mean (SD)	5618 (7387)	4613 (6007)	<0.0001	4540 (6765)	4416 (5944)	0.6724
Median (P10–P90)	3014 (495–14,070)	2516 (568–11,657)		2333 (452–10,863)	2283 (448–11,180)	
Epilepsy-related direct health care costs in the 1-year pre-index period, £						
Mean (SD)	997 (2361)	633 (1965)	<0.0001	804 (2134)	726 (2201)	0.4314
Median (P10–P90)	42 (0–2797)	11 (0–2188)		23 (0–2599)	11 (0–2599)	
Health care resource use per patient in the 1-year pre-index period Mean (SD) number of:						
GP practice consultations	35.13 (29.55)	43.69 (36.33)	<0.0001	36.48 (31.92)	37.04 (31.15)	0.6973
A&E visits	0.83 (1.26)	0.70 (1.28)	0.0003	0.72 (1.41)	0.70 (1.15)	0.6173
Outpatient non-A&E referrals	0.96 (1.45)	1.27 (1.40)	<0.0001	1.08 (1.35)	1.08 (1.29)	0.9446
Inpatient hospitalizations	1.64 (5.63)	1.20 (2.70)	0.0004	1.44 (7.27)	1.22 (3.41)	0.4126
Hospitalization duration, mean (SD), days						
	6.96 (19.70)	3.64(11.89)	<0.0001	4.30 (11.32)	4.09 (11.48)	0.6873

*Abbreviations: A&E* accident and emergency, *EIAED* enzyme-inducing antiepileptic drug, *GP* general practitioner, *nEAAED* non-enzyme–active antiepileptic drug, *P10–P90* 10th to 90th percentile, *SD* standard deviation. ^a^ *t* test

After matching, each cohort consisted of 951 patients and baseline characteristics were similar for all available potential confounders (Tables 1–3).

### Time to cohort treatment failure and index AED failure

**Fig. 1:**
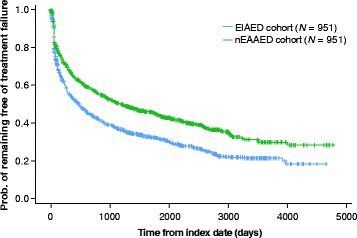
Time to cohort treatment failure in the matched cohorts. *EIAED* enzyme-inducing antiepileptic drug, *nEAAED* non-enzyme–active antiepileptic drug

In the matched populations, the proportion of patients remaining in follow-up after 1 year was smaller for the EIAED cohort than the nEAAED cohort (49.1% vs 60.6%). The median time to cohort treatment failure (allowing within-group AED switching) was 468 days in the EIAED cohort compared with 1194 days in the nEAAED cohort, with a total follow-up time of 2297 vs 2881 years, respectively (Fig. 1). Index AED failure (discontinuation of index AED, switch or addition of another) occurred in 68.3% of patients in the matched EIAED cohort and 62.7% of patients in the matched nEAAED cohort, with a median time to index AED failure of 452 days vs 869 days, respectively. Index AED failure owing to discontinuation of the index AED occurred in 41.3% of the EIAED cohort and 43.7% of the nEAAED cohort. Most discontinuations occurred during the first 3 months of treatment in both cohorts. Treatment failure owing to an addition of another AED occurred in 27.0% of the EIAED cohort and 18.9% of the nEAAED cohort.

### All-cause direct health care cost

**Fig. 2:**
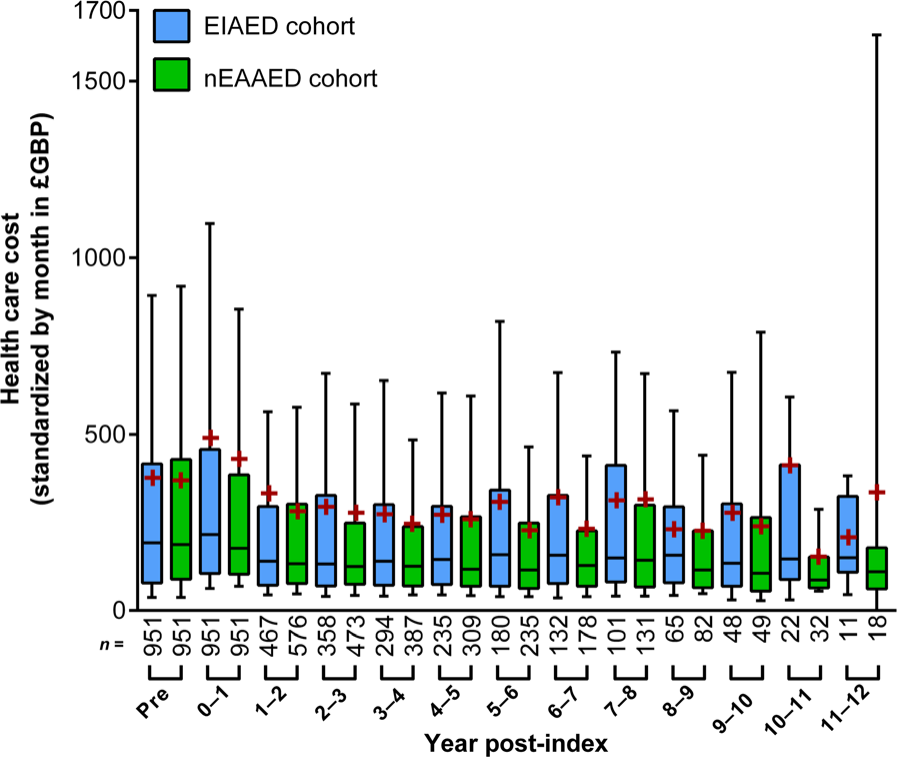
Standardized monthly all-cause direct health care cost in each post-index year for the matched cohorts. Whiskers extend from the 10th to the 90th percentile; boxes extend from the 25th to the 75th percentile; center line is the median; red cross is the mean. *EIAED* enzyme-inducing antiepileptic drug, *nEAAED* non-enzyme–active antiepileptic drug

The median (range) standardized monthly direct health care cost over the entire follow-up period was £229 (£18–£18,613) for the EIAED cohort and £188 (£16–£33,880) for the nEAAED cohort (*p* = 0.0091; Table 4). Higher median monthly health care cost was observed in the EIAED cohort in every year post-index, for all patients who *started* the year in question (Fig. 2). The difference between the cohorts became consistently greater from year 3. When considering yearly cost for patients *completing* each year of follow-up, the median yearly direct health care cost was higher in the EIAED cohort than the nEAAED cohort in every year except year 2 (£53 difference). Similar findings were also observed when cost was calculated only for patients who still had an active match pairing at the start of the year.

**Table 4:** Standardized monthly direct health care cost over the follow-up period

Costs are in 2014 £GBP		EIAED (*n* = 951)	nEAAED (*n* = 951)
Total direct all-cause health care costs	Median (range)	229 (18–18,613)	188* (16–33,880)
	Mean (SD)	495 (1016)	432 (1272)
A&E visits	Median (range)	0 (0–1740)	0 (0–316)
	Mean (SD)	9 (64)	6 (22)
AED medications	Median (range)	9 (2–1407)	8 (2–438)
	Mean (SD)	23 (58)	13 (22)
GP practice consultations	Median (range)	114 (12–2816)	102 (8–1591)
	Mean (SD)	161 (187)	143 (146)
Inpatient hospitalizations	Median (range)	0 (0–13,337)	0 (0–33,394)
	Mean (SD)	236 (830)	217 (1203)
Non-AED medications	Median (range)	7 (0–797)	6 (0–939)
	Mean (SD)	30 (69)	28 (71)
Outpatient, non-A&E referrals	Median (range)	1 (0–489)	2 (0–284)
	Mean (SD)	16 (43)	9 (22)
Test procedures	Median (range)	7 (0–761)	7 (0–518)
	Mean (SD)	20 (43)	16 (32)
Total epilepsy-related direct health care costs	Median (range)	27 (3–15,781)	21 (2–3942)
	Mean (SD)	124 (608)	87 (287)

*Abbreviations: A&E* accident and emergency, *AED* antiepileptic drug, *EIAED* enzyme-inducing antiepileptic drug, *GP* general practitioner, *nEAAED* non-enzyme–active antiepileptic drug, *SD* standard deviation

*p = 0.0091 vs EIAED, calculated by Mann–Whitney *U* test

### Specific health care resource use and cost

Patients in the matched EIAED cohort had more GP practice consultations (mean standardized per month [standard deviation], 5.39 [5.87] vs 4.71 [4.62]) and outpatient referrals (0.12 [0.29] vs 0.07 [0.16]), than those in the matched nEAAED cohort. Health care cost standardized by month were higher in the EIAED cohort for every resource category (costs of drugs [AEDs and other medications], GP consultations, test procedures, A&E visits, inpatient hospitalizations and outpatient referrals and procedures) (Table 4). The median standardized monthly cost of AED medication was £9 and £8 in the EIAED and nEAAED cohorts, respectively. The mean cost made up 4.6% and 3.0% of the total mean direct health care cost.

### Epilepsy-related direct health care cost

The median standardized monthly direct epilepsy-related cost (range) was £27 (£3–£15781) for the matched EIAED cohort and £21 (£2–£3942) for the matched nEAAED cohort. Over the entire follow-up period, a larger percentage of the mean direct health care cost was epilepsy-related in the EIAED cohort than the nEAAED cohort (25.1% vs 20.1%; Table 4). The mean number of epilepsy-related visits per month over the follow-up period was numerically higher in the EIAED cohort in all categories except GP consultations. Over the entire follow-up period, the proportions of patients requiring epilepsy-related A&E visits, GP consultations and inpatient hospitalization were slightly higher in the nEAAED cohort compared with the EIAED cohort; however, these latter rates are not adjusted for differing follow-up time.

### Incident comorbidities and concomitant medications

During the follow-up period, incident (new) comorbidities diagnosed in ≥ 5.0% of the patients in either cohort included soft tissue disorders, essential hypertension, respiratory infections, back pain, hypertension, joint disorders, urinary system disorders, convulsions, falls, disorders of skin and subcutaneous tissue, otitis externa, depression, abdominal and pelvic pain and conjunctivitis (Table S2).

Incidence rates were estimated using the number of patients with at least one new diagnosis during the post-index period (and no diagnosis for the respective comorbidity during the pre-index period) divided by the total cohort follow-up time in years (Table S2). Rate ratios were calculated for the two cohorts. Differences between the cohorts were observed in both directions (higher and lower), and no relationship to exposure was observed. However, many of the common (≥5.0%) incident comorbidities were reported more frequently in the EIAED cohort than in the nEAAED cohort, including convulsions (1.43 times as frequently), otitis externa (1.61 times), essential hypertension (1.31 times), nonspecified fall (1.21 times), upper respiratory infections (1.20 times), other skin disorders (1.19 times) and abdominal and pelvic pain (1.05 times). Others were slightly more frequent in the nEAAED cohort: dorsalgia, joint disorders and injuries of unspecified body region (1.07, 1.11 and 1.22 times, respectively).

Among the less common (< 5.0%) incident comorbidities, the following were reported at a markedly higher frequency in the EIAED cohort: fracture of foot, forearm and lumbar spine (7.33, 4.64 and 4.40 times as frequently), other metabolic disorders and manic episode (4.33 and 4.33 times as frequently). Some other comorbidities were more frequently reported in the nEAAED cohort: complications and ill-defined descriptions of heart disease, and ulcerative colitis (9.75 and 4.25 times as often, respectively).

The profiles of new medications prescribed during post-index period (and not prescribed during the pre-index period) were generally similar in the matched cohorts. The most frequently prescribed drug classes (other than AEDs) were antibacterial drugs (39.0% in the EIAED cohort vs 43.8% in the n-EAAED cohort), analgesics (23.0% vs 31.0%), antisecretory drugs (16.5% vs 19.2%) and topical corticosteroids (22.5% vs 23.5%).

Among all drug classes, thyroid and anti-thyroid drugs, mucolytics, anti-diabetics and local anaesthetics/anti-pruritics had new prescription rates 3.73-, 2.26-, 2.14- and 2.13-fold more in the EIAED cohort than nEAAED cohort, respectively. Topical circulatory preparations, antiperspirants and preparations for warts/calluses had a new prescription rate 6.00-, 5.25- and 4.00-fold more in the nEAAED cohort than in the nEAAED cohort, respectively.

## Discussion

This study evaluated up to 12 years of retrospective medical records from patients initiating AED therapy in the UK. Our unmatched data showed that during the study period (2001–2011), patients prescribed EIAEDs as initial AED therapy were older, more likely to be male and had more comorbidities than those prescribed nEAAEDs. These differences were, in turn, reflected in higher health care resource use and cost, comorbidity and concomitant medication use in the unmatched EIAED cohort for the pre-index year. The older age and lower proportion of female patients initiating therapy on an EIAED may be justified by the risks of reproductive hormone abnormalities and interactions with hormonal contraceptives associated with EIAED use [2, 12]. However, our findings remain somewhat surprising, as patients with pre-existing comorbidities and concomitant medications are generally less suited to receiving EIAEDs, owing to their potential for drug-drug interactions. This would suggest that EIAED prescribing was not optimally targeted during the assessed period.

Our findings suggest that after controlling for baseline differences between the cohorts by propensity score matching, the median total direct health care cost (standardized per month) associated with the use of EIAEDs was higher than with nEAAEDs. This study was the first to assess the relative direct cost of EIAED vs nEAAED therapy over a clinically relevant period of time. Our results suggest that the higher cost observed for the EIAED cohort was related to higher health care utilization in general. We were unable to identify any specific comorbidities linked to the higher observed costs, which might partly be due to limitations of the coded source data. The EIAED cohort showed higher AED costs, which might have been unexpected, as the acquisition cost of the newer nEAAEDs is usually higher. The larger AED costs could be related to the more frequent need for polytherapy observed in the EIAED cohort, demonstrated by the higher proportion of patients who added another AED and the shorter time on index AED monotherapy. Analyses of matched patient cohorts showed a shorter time to cohort treatment failure, and to index AED failure, for the EIAED cohort vs the nEAAED cohort. Although these findings might suggest poorer long-term clinical outcomes associated with EIAED therapy, the CPRD contains limited seizure frequency data, so we are unable to investigate these suggestions. Unfortunately, the reasons for AED failure are not readily available in the data source.

Cost analyses using CPRD data are uncommon. The major advantage of CPRD data over other patient record databases (such as US insurance claims databases) is that it offers a unique source of long-term, continuous patient data. The last estimate for the direct costs of epilepsy in the UK was published 22 years ago. Cockerell *et al.* [13] evaluated an average of 6.6 years of health care utilization data from up to 1195 UK patients, collected as part of the National General Practice Study of Epilepsy [14]. The cost of epilepsy to a newly diagnosed individual with epilepsy was estimated to be £611 in the first year, decreasing to £221 in year 2 and to £169 by year 8. Declining costs after the first year were also observed in other costing studies [15, 16]. These decreasing costs over time are dissimilar with our findings. A large part of the observed difference is likely to be due to methodology and study period, and results can therefore only be reliably compared within studies. The methodological challenges of costing epilepsy is discussed in detail by Cockerell et al. [13].

The profile of index AED prescription rates reflected the evolving treatment guidelines, AED availability and physician preferences over the 10-year selection period; nEAAEDs were increasingly preferred as the selection period progressed. In the UK, the National Institute for Health and Care Excellence (NICE) assesses the value of treatment options based on analyses of outcomes and costs, publishing treatment recommendation guidelines to actively influence the selection of interventions. The guidelines recommend that patients with epilepsy initiate AED treatment as monotherapy; however, a preference for EIAED or nEAAED has not been explicitly made. Later generation AEDs (mostly nEAAEDs) tend to gain market approval initially as adjunctive therapy, with a monotherapy indication added later. This leads to a lag in treatment availability for newly diagnosed patients [17]. The peak year for patient indexing in the EIAED cohort was 2004, which is consistent with the 2004 NICE guidelines supporting carbamazepine as a first-line epilepsy treatment (along with phenytoin, valproic acid and divalproex) [18]. For the nEAAED cohort, the peak year for patient indexing was 2010, which may be indicative of the changing prescribing attitudes following the publication of the 2007 SANAD study, which showed lamotrigine to be more clinically and cost-effective than carbamazepine [19]. Evidence from SANAD contributed to the 2012 update of the NICE guidelines [6], which recommend lamotrigine, alongside four other AEDs with differing enzyme induction properties (carbamazepine, levetiracetam, oxcarbazepine and sodium valproate), as a first-line treatment for patients with focal epilepsy. Mild enzyme inducing AEDs were not included in either cohort or allowed during follow-up. Given these AEDs’ mild enzyme induction profile, classifying them as either EIAEDs or nEAAEDs, or allowing their use in follow-up, would have potentially biased conclusions regarding cost outcomes between EIAEDs and nEAAEDs. Based on our data it is therefore not possible to make inferences regarding the cost outcomes associated with mild enzyme inducers and how they compare with those of EIAEDs or nEAAEDs.

The nature of incident comorbidity diagnoses during the follow-up period was generally similar between matched cohorts. Differences between the cohorts were observed in both directions (higher and lower). New prescriptions for several types of drugs were less common in the EIAED cohort during the follow-up, and this may be indicative of the increased risk of drug interactions, leading to caution when selecting concomitant drug therapies. This is also potentially reflected in the shorter time to end of follow-up in the EIAED cohort, as patients may require relevant treatments that are not well suited for concomitant therapy with EIAEDs. Overall, findings relating to the incidence of new comorbidities or prescriptions were not clearly related to AED exposure or type. This may have been due to the limitations of using coded data.

A particular strength of our study was the patient matching by propensity score, which utilized several hundred available demographic and clinical characteristics. By eliminating known and observable confounding factors, patient matching allowed an accurate estimation of the cost and time to treatment failure associated with EIAED vs nEAAED therapy to be made in a generally representative population of previously untreated epilepsy patients. However, not all potential confounding factors could be controlled for in this analysis. For example, regional differences in the standard of epilepsy care have been noted in the UK [20, 21], and may relate to personal preferences of the GP, practice type, size, location and local formulary access to certain AEDs. There is also a known correlation between social deprivation and increased epilepsy prevalence [22]. Additional relevant factors may have been excluded from the propensity scoring owing to lack of information in CPRD data (e.g. seizure type and frequency). As such, there is the chance that the results are biased by residual, unmeasured confounding.

To obtain the most accurate costings, we attempted to match each health care resource used with the most up-to-date cost information in 2014 GBP. This approach was taken because of the long follow-up period in our study, and allowed comparison between the cohorts of patients entering the study longitudinally. Costs for each health care resource used were calculated from published unit costs and are as accurate as possible given the data available. The study’s primary variable summed the value of every health care resource used over the entire follow-up period. When considering cost analyses over time, we chose to look at the standardized monthly cost per year for all patients starting the year in question. This had the benefit that cost data from patients who left follow-up during the year (following treatment success or failure) were reflected accurately in the analysis. Sensitivity analyses showed that the cost trends and cohort comparisons were similar when cost was only analysed for patients completing the year, and when considering only those patients with an active match at the start of the year. Cost analyses are nearly always estimates with inherent inaccuracies, and all electronic medical record and billing data are subject to some level of miscoding. The AED exposure in this study was based on sometimes incomplete prescription duration data. Initial assessments found structured duration information to be missing in over a quarter of individual AED prescriptions. Therefore, a novel algorithm was used to determine duration for each prescription, as described in the methods. The most likely maximum duration of each AED prescription was calculated as twice the median duration for all prescriptions of that type (type defined based on generic name, dose and package size) in the entire study database. Twice the median was chosen as the likely maximum duration in order to avoid underestimating a patient’s exposure to an AED; since discontinuing an AED usually requires a slow down-titration, a longer treatment duration is typically associated with the last prescription. The end of exposure to an AED was defined as the start date of an exposure gap longer than four times the imputed maximum duration of the last prescription. This ‘allowed gap’ may appear liberal but was chosen to avoid ending exposure too soon in case of missing data – the assumption being that in case of a ‘long’ gap followed by a ‘new’ prescription of the same AED, it is clinically unlikely that there really was a gap. These miscoding issues and imputation assumptions are not expected to differ between cohorts and therefore do not lead to bias.

Our analyses are retrospective and descriptive in nature, and findings cannot be directly extrapolated to current clinical practice, as prescription behaviour may have changed according to guidelines. There were several other limitations in our study regarding information availability, such as the lack of seizure data, reasons for treatment discontinuation and adverse event reporting. While our results indicate increased health care resource use associated with the use of EIAEDs in epilepsy, the direct cause of this use, beyond its relation to epilepsy or otherwise, needs further investigation and will suffer from the lack of information noted above. Epilepsy comprises a diverse group of disorders and our analysis was conducted on a potentially diverse pool of patients; therefore, it might not accurately reflect patients with distinct subtypes of epilepsy (i.e. syndrome, localization or aetiology), which was found to be poorly coded in CPRD data. Furthermore, events occurring before the 1-year pre-index period were not included in the analysis. Taken together, there is a possibility that specific patient subgroups are erroneously included or omitted; for example, inclusion of those prescribed AEDs for indications other than epilepsy, and inclusion of patients misdiagnosed with epilepsy.

## Conclusions

Our analysis of UK CPRD data suggests that during the studied period, EIAEDs were prescribed as an initial epilepsy therapy to older patients, who were more likely to be male and had higher baseline health care resource use and cost than patients prescribed nEAAEDs. Given the risks associated with enzyme induction, this prescribing pattern appears to be suboptimal. In long-term, matched cohort analyses, a higher average total direct health care cost and a shorter time to treatment failure were associated with EIAED vs nEAAED therapy. We conclude that changing current treatment practices could potentially improve patient outcomes and reduce health care costs.

## Additional file

Additional file 1: Further details on the calculation of health care resource use and costs. **Table S1.** Variables on which the cohorts were significantly different at baseline. **Table S2.** Most common incident diagnoses during the post-index period. (DOCX 26 kb)

## Abbreviations

A&E: accident and emergency
AED: antiepileptic drug
CPRD: Clinical Practice Research Datalink
CYP450: cytochrome P450
EIAED: enzyme-inducing antiepileptic drug
GP: general practitioner
HES: Hospital Episode Statistics
nEAAED: non-enzyme-active antiepileptic drug
NICE: National Institute for Health and Care Excellence
