# Supplementary material for: Health care cost associated with the use of enzyme-inducing and non-enzyme–active antiepileptic drugs in the UK: a long-term retrospective matched cohort study
Source: BMC Neurol. 2017 Mar 23;17:59. doi: 10.1186/s12883-017-0837-y (PMC5364597; doi:10.1186/s12883-017-0837-y)
Supplement: Additional file 1: — Further details on the calculation of health care resource use and costs. Table S1. Variables on which the cohorts were significantly different at baseline. Table S2. Most common incident diagnoses during the post-index period. (DOCX 26 kb) [file 12883_2017_837_MOESM1_ESM.docx]

**Methods**

**Health care resource use and costs**

Medication costs were calculated per prescription, using the substance name, and April 2014 published prescription costs [1]. General practitioner consultations, including telephone consultations, were identified through the primary care data. Costs were obtained from the 2013 Unit Costs of Health and Social Care [2] and adjusted for assumed duration and type of consultation. Costs for test procedures were based on NHS reference data. Outpatient non–accident and emergency (A&E) visits were identified through the referrals table in the primary care data and costed per specialty, using 2012/13 references [3]. If no specialty was entered, it was assumed to be “general surgery.” A&E visits were identified using the referrals table in the primary care data, or as inpatient hospitalizations through A&E in Hospital Episode Statistics data. Based on 2012/2013 reference costs [3], each A&E visit was costed as £116.02 in 2014 £GBP. Inpatient hospitalizations were identified from Hospital Episode Statistics data, and costs were assigned based on the types of intervention and diagnoses according to the UK NHS Payment by Results system [4]. Each cost item was adjusted to 2014 £GBP using ratios given in the Hospital and Community Health Services pay and price inflation index [5]. Resource use and associated cost were deemed epilepsy-related if accompanied by a diagnosis of epilepsy.

**References**

1. NHS Business Services Authority. Prescription Cost Analysis (PCA) data. http://www.nhsbsa.nhs.uk/PrescriptionServices/3494.aspx. Accessed Q1 2015.

2. PSSRU Personal Social Services Research Unit. Unit costs of health and social care 2013. http://www.pssru.ac.uk/project-pages/unit-costs/2013/index.php. Accessed Q1 2015.

3. GOV.UK. Policy paper: NHS reference costs 2012 to 2013. https://www.gov.uk/government/publications/nhs-reference-costs-2012-to-2013. Accessed Q1 2015.

4. NHS England. NHS payment system. https://www.england.nhs.uk/resources/pay-syst/. Accessed Q1 2015.

5. PSSRU Personal Social Services Research Unit. Unit costs of health and social care 2014. http://www.pssru.ac.uk/project-pages/unit-costs/2014/index.php. Accessed Q1 2015.

**Table S1** Variables on which the cohorts were significantly different at baseline

| Covariates that were significantly different between the EIAED and nEAAED cohorts before the propensity matching model was applied | |
| --- | --- |
| Variable | Scored as |
| Age at index date | ($x$+1) / $x$ |
| Index year (rescaled 0–9) |  |
| Years since most recent epilepsy diagnosis |  |
| Number of GP practice consultations |  |
| Gender | Male vs Female |
| Epilepsy type | Partial vs Unspecified |
| Index AED was first ever AED | Yes vs No |
| Took Analgesics |  |
| Antiarrhythmic drugs |  |
| Antibacterial drugs |  |
| Anticoagulants |  |
| AEDs |  |
| Antiprotozoal drugs |  |
| β-adrenoceptor blocking agents |  |
| Drugs used for diabetes |  |
| Had A&E visits |  |
| Outpatient (non-A&E) referrals |  |
| Acute nasopharyngitis (common cold) |  |
| Anemias or other blood disorders |  |
| Arterial embolism and thrombosis |  |
| Benign neoplasm of the meninges |  |
| Care involving dialysis |  |
| Coxarthrosis (arthrosis of the hip) |  |
| Dorsalgia |  |
| Down syndrome |  |
| Hemorrhoids |  |
| Intracranial injury due to external causes |  |
| Malaise and fatigue |  |
| Malignant neoplasm of the brain |  |
| Malignant neoplasm of the breast |  |
| Mental and behavioral disorders due to the use of alcohol |  |
| Mental and behavioral disorders due to the use of tobacco |  |
| Nondiagnosed abnormal involuntary movements |  |
| Nonspecified joint derangements |  |
| Nonspecified polyneuropathies |  |
| Nonspecified pleural effusion |  |
| Nonspecified spondylopathies |  |
| Otitis externa |  |
| Pain in the throat or chest |  |
| Special screening examination for nonspecified  disease and disorders |  |
| Streptococcus and staphylococcus as the cause of a previous disease |  |
| Stroke (not specified as hemorrhage or infarction) |  |
| Surgical operation/procedure due to abnormal reaction or later complication, without mention of misadventure |  |
| Ventral hernia |  |
| Visual disturbances |  |

*Abbreviations: A&E* accident and emergency, *AED* antiepileptic drug, *EIAED* enzyme-inducing antiepileptic drug, *GP* general practitioner, *nEAAED* non-enzyme–active antiepileptic drug

Statistical difference was assessed by *t* test, chi-square test or Fisher’s exact test, as appropriate

Of 318 baseline variables, only those with an incidence of >1 % and were significantly different between the unmatched EIAED and nEAAED were included in the propensity mode. Variables were assessed in the pre-index year and included in the table if significantly different in the model

**Table S2** Most common incident diagnoses during the post-index period

|  | EIAED | | | nEAAED | | |
| --- | --- | --- | --- | --- | --- | --- |
|  | Most common (≥ 5.0 %) “new” diagnoses occurring during the post-index period, in either cohort | | | | | |
|  | % patients reporting | % patients as new diagnoses | Incidence rate of new cases^a^ | % patients reporting | % patients as new diagnoses | Incidence rate of new cases^a^ |
| Nonspecified soft tissue disorders^b^ | 15.5 | 13.7 | 5.65 | 20.3 | 17.7 | 5.64 |
| Essential hypertension | 13.9 | 9.1 | 3.75 | 14.1 | 8.7 | 2.86 |
| Acute lower respiratory tract infection | 11.9 | 10.2 | 4.13 | 14.7 | 13.2 | 4.27 |
| Back Pain (dorsalgia) | 9.8 | 8.8 | 3.57 | 13.1 | 11.8 | 3.81 |
| Personal history of certain other diseases | 9.8 | 6.8 | 2.80 | 12.4 | 9.3 | 3.03 |
| Acute upper respiratory tract infection | 8.8 | 8.5 | 3.50 | 9.1 | 8.8 | 2.91 |
| Nonspecified disorders of urinary system | 8.4 | 6.0 | 2.47 | 10.2 | 8.2 | 2.65 |
| Nonspecified convulsions | 8.4 | 6.7 | 2.81 | 7.9 | 5.7 | 1.97 |
| Nonspecified joint disorders | 6.8 | 6.7 | 2.76 | 9.7 | 9.3 | 3.05 |
| Nonspecified falls | 6.8 | 6.2 | 2.55 | 6.5 | 6.4 | 2.1 |
| Disorders of lipoprotein metabolism and other lipidemias | 6.3 | 4.9 | 2.05 | 6.0 | 4.7 | 1.55 |
| Nonspecified diseases of the skin and subcutaneous tissue | 5.8 | 5.6 | 2.31 | 6.4 | 5.9 | 1.94 |
| Candidiasis | 5.6 | 5.0 | 2.07 | 5.8 | 5.7 | 1.86 |
| Abdominal and pelvic pain | 5.5 | 4.5 | 1.83 | 5.6 | 5.3 | 1.75 |
| Depressive episode | 5.2 | 4.7 | 1.94 | 6.7 | 6.0 | 1.95 |
| Otitis externa | 5.3 | 5.0 | 2.06 | 4.5 | 3.9 | 1.28 |
| Nonspecified dermatitis | 4.6 | 4.7 | 1.93 | 5.8 | 5.7 | 1.86 |
| Conjunctivitis | 4.2 | 3.6 | 1.46 | 6.3 | 5.9 | 1.95 |
| Dermatophytosis | 4.0 | 3.8 | 1.59 | 5.7 | 5.8 | 1.9 |
| Injury of unspecified body region | 3.5 | 3.4 | 1.44 | 5.5 | 5.3 | 1.76 |

*Abbreviations: EIAED* enzyme-inducing antiepileptic drug, *nEAAED* non-enzyme–active antiepileptic drug

^a^Incident rate is number of patients with at least one diagnosis during the post-index period, who had no diagnosis for the respective comorbidity during the pre-index period, divided by the total cohort follow-up time in years

^b^Nonspecified soft tissue disorders included *International Classification of Disease, Tenth Revision* codes, for rheumatism (unspecified), myalgia, neuralgia and neuritis (unspecified), panniculitis (unspecified), hypertrophy of (infrapatellar) fat pad, residual foreign body in soft tissue, pain in limb, fibromyalgia, other specified soft tissue disorders, soft tissue disorder (unspecified)

New prescriptions denote patients who had no diagnosis for the comorbidity in the 1-year pre-index period
